# Microbial community compositions in breast implant biofilms associated with contracted capsules

**DOI:** 10.1371/journal.pone.0249261

**Published:** 2021-04-08

**Authors:** Sean A. Crowe, Rachel L. Simister, Jenifer S. Spence, Paul A. Kenward, Aaron C. Van Slyke, Peter Lennox, Nick Carr

**Affiliations:** 1 Department of Microbiology and Immunology, University of British Columbia, Vancouver, BC, Canada; 2 Department of Surgery, University of British Columbia, Vancouver, BC, Canada; 3 Vancouver Plastic Surgery, Vancouver, BC, Canada; 4 Skinworks, Vancouver, BC, Canada; University of Illinois, UNITED STATES

## Abstract

Subclinical bacterial infections (biofilms) are strongly implicated in breast augmentation failure due to capsular contracture, and while these infections are generally ascribed to common skin commensals, this remains largely unsubstantiated through robust cultivation independent analyses. To determine capsule biofilm microbial community compositions, we employed amplicon sequencing of the 16S rRNA gene using DNA extracted from breast implant capsule samples. These cultivation independent analyses revealed that capsule associated biofilms are more diverse than canonical single-species infections, but have relatively low diversity (~ <100 species) compared to many host-associated microbial communities. In addition to taxa commonly associated with capsular contracture, the biofilms analyzed comprised a number of taxa that escaped detection in cultivation-dependent work. We have also isolated several key taxa identified through the culture-independent analyses. Together our analyses reveal that capsule biofilms are more diverse than cultivation studies suggest and can be heterogeneous within an individual capsule, between breasts of the same patient, across similar implant types, and over a range in severity of contracture. The complex nature of these communities requires further study across a broader suite of patients in addition to higher resolution analyses including metagenomics to better assess the fundamental role of microorganisms in capsular contracture.

## Introduction

The human body is comprised of more than one microbial cell for every human cell [1] and these microbial cells, the human microbiome, are both taxonomically and physiologically diverse. The metabolic blueprint that codes for healthy human physiology is distributed across more than 2000 Mb (assuming more than 1000 species with mean genome sizes of 2 Mb) of non-redundant microbial genomic information in addition to the 3200 Mb of the human genome [2]. This hidden majority of microbial cells and genomic information often plays an outsized role in human health impacting inflammation, immunity, and a vast array of acute and chronic human health issues [3–6]. Considerable efforts are underway to map the diversity, metabolic potential, and ecological principles that define interactions within the human microbiome and between the microbiome and the human host to inform translational outcomes across the health sector [7–9].

Illness related to microbe-host interactions (dysbiosis) is best thought of as a disruption to the composition and activity of the healthy microbiome. Surgical procedures, including implantation, often cause dysbiosis as a result of immune responses, introduced microbial community members, or the alteration of the physical-chemical properties of the tissue environment. Colonization of surgical implants by microorganisms, both innate and foreign, and the subsequent development of microbial biofilms on implant surfaces often leads to complications including inflammation and other immune responses, implant failure, or disruption of optimal wound healing and scar tissue formation [10]. Prevention of implant biofilm formation currently relies on a range of poorly informed interventions including treatment with antibiotics of varying specificity that target putative biofilm community members [11–13]. Historically, these community members have been identified through cultivation dependent methodologies, despite cultivation being notoriously biased against key community members that remain undetected [14], thereby confounding interventions intended to improve health. New cultivation-independent methods overcome these biases and yield a more complete and accurate description of relevant microbial community members [15] and their potential physiology and function [16]. As these approaches are increasingly applied to the microbiome, we are gaining an appreciation for the roles different community members play in healthy and unhealthy tissue-microbiome functioning [17]. Cultivation-independent approaches remain under-applied, however, to the vast array of surgical implant complications that plague the medical community.

All implanted foreign bodies induce a host reaction consisting of production of a capsular layer that surrounds the foreign material. In the case of breast implants, the capsule can vary from soft and pliable (Baker grade I) to a pathologic contracted layer causing the implant to become distorted, hard, and painful (Baker grade IV) [12, 18–20]. Capsular contracture is the most common complication following breast augmentation surgeries_,_ and these are among the most widely performed surgical implantations. Subclinical bacterial infections (biofilms) are strongly implicated in capsular contracture [21, 22], and while these infections are generally ascribed to common skin commensals, this remains largely unsubstantiated through robust cultivation-independent analyses. Notably, capsular contracture and associated biofilm formation are now also potentially linked to Breast Implant Associated Anaplastic Large Cell Lymphoma (BIA_ALCL) [23–25]. While capsular contracture represents a critical human health challenge, conventional preventative measures such as irrigation with antibiotics remain only partially effective in its prevention, in part because the bacteria responsible for biofilms are highly resistant to many antibiotics [26]. New preventative measures are, therefore, needed to reduce the incidence of this complication [27, 28].

*Staphylococcus epidermidis* has been implicated in biofilm formation on implant capsules based on its recovery in standard culture dependent medical studies [29], as well as related *Staphylococcus* taxa in newer culture-independent studies [30, 31]. *S*. *epidermidis* is classically considered non-pathogenic, though it is increasingly associated with sub-clinical infections, in particular those involving prosthetic devices [32–34]. While *Staphylococcus sp*. almost certainly play a role in the formation of capsular biofilms [35, 36], they likely do so within a broader ecological context defined by other community members that evade detection through cultivation, as indicated through culture independent analyses [30, 31]. Detailed information on the composition of the microbial communities from contracted capsules is thus a much-needed first step towards diagnosing the causative microbial agents in capsular biofilm formation and innovating new solutions for prevention and treatment of capsular contracture. Our combined cultivation-dependent and independent approach reveals that biofilms from pathological and non-pathological contracted capsules comprise diverse microbial communities, including many members that were undetected in cultivation-based studies.

## Results

High quality DNA was recovered from all samples, but some samples (Table 1) did not yield amplicons of either bacterial or archaeal 16S rRNA genes, even through nested PCR amplification. Microbial DNA was successfully detected, defined as a positive amplification of the 16S rRNA gene, in capsules from 14 of 17 patients, or in more than 80% of cases. Bacterial loads were quantified through qPCR of the 16S rRNA gene and ranged from 1 x 10^5^ to 5 x 10^7^ copies g^-1^ of tissue extracted (Table 1). We also tested for inhibition of gene amplification commonly observed when the target template is strongly diluted by non-target DNA or by other organic compounds (Table 2). These tests reveal that our qPCR results may underestimate total 16S rRNA gene copies by as much as a factor of 100. Our qPCR-based estimates of bacterial loads should thus be considered minimum loads. Corresponding DNA extraction blanks ranged from 10^2^ to 10^3^ copies g^-1^, assuming a nominal extraction mass of 0.2 g. The abundance of bacterial 16S rRNA gene copies recovered from capsule and tissue was, therefore, at least 2 orders of magnitude higher than that present in the reagents used in DNA extraction, purification, and PCR, and we therefore concluded that contamination from extraction was negligible.

**Table 1:** Capsular samples, qPCR quantification of the capsular tissue microbial load (gene copies per gram of tissue), respective number of sequences analyzed, observed sequences, chao 1 alpha diversity indices, implant type, and Baker scale rating.

Sample ID	qPCR (copies/g)	Total number of OTUs	Chao1	Implant Type	Implant Plane	Baker Scale Grade	Aesthetic v Recon (A v R)	Yrs in place	Age range (decade) at last surgery	BMI	Smoker	Reason for Surgery	Notes
C1L Rep. 1†	4.33E+05	80	97	TAG- 410	Retro.	1	R	8	70–80	16	N	Malrotation	Non-adherent
C1L Rep. 2†	3.87E+05	83	135										
C1L Rep. 3†	7.73E+05	134	138										
C1L Rep. 4†	5.02E+05	137	105										
C1L Rep. 5†	4.51E+05	93	98										
C1L Rep. 6†	6.08E+05	92	94										
C1R†	1.11E+06	38	61	TAG- 410	Retro.	1	R	8	70–80	16	N	Malrotation	Non-adherent
C2L	5.18E+05	70	78	TAG- 410	Retro.	1	A	5	40–50	24	N	Pain / Malrotation	Partially-adherent
C2R Rep. 1	1.99E+07	21	37	TAG- 410	Retro.	1	A	5	40–50	24	N	Pain / Malrotation	Non-adherent
C2R Rep. 2	5.64E+05	45	107										
C2R Rep. 3	2.28E+07	48	78										
C2R Rep. 4	1.79E+07	43	141										
C2R Rep. 5	2.09E+07	16	42										
C3L	NA	NA	NA	TAG	Retro.	1	A	7	40–50	21	N	Right rupture (intracapsular)	Non-adherent IR
C3R	NA	NA	NA	TAG	Retro.	1	A	7	40–50	21	N	Right rupture (intracapsular)	Non-adherent IR
C4L	6.38E+05	79	98	SRG	Sub.	4	A	35	50–60	24	N	Cap con / Bilateral rupture (Extracapsular)	Bilateral ER
C4R Rep. 1	5.75E+05	31	88	SRG	Sub.	4	A	35	50–60	24	N	Cap con / Bilateral rupture (Extracapsular)	Bilateral ER
C4R Rep. 2	5.14E+05	131	242										
C4R Rep. 3	5.77E+05	71	55										
C4R Rep. 4	5.64E+05	78	103										
C4R Rep. 5	5.64E+05	112	255										
C5L	4.19E+05	27	34	SRS	Retro.	3	A	2	30–40	21	N	Ptosis	-
C6L	NA	NA	NA	SRS	Retro.	2	A	15	50–60	18	N	Ptosis	
C6R	NA	NA	NA	SRS	Retro.	2	A	15	50–60	18	N	Ptosis	
C7 Rep. 1	9.03E+05	47	54	TAG- 410	Retro.	4	A	7	40–50	24	N	Cap con	Partially-adherent double capsule
C7 Rep. 2	4.51E+05	43	24										
C7 Rep. 3	1.11E+06	43	62										
C7 Rep. 4	3.21E+06	69	72										
C7 Rep. 5	3.58E+06	60	107										
C8R	1.14E+07	79	-	SRS	Retro.	1	A	12	30–40	20	N	Size change / Mastopexy	-
C9L	1.80E+06	65	80	TRG	Retro.	2	A	4	30–40	20	N	Left peri-prosthetic fluid	-
C9R	3.06E+05	58	72	TRG	Retro.	2	A	4	30–40	20	N	Left peri-prosthetic fluid	-
C10L	NA	NA	NA	SRG	Retro.	4	A	36	80–90	19	Y	Bilateral rupture (intracapsular)	Partially-adherent
C10R	NA	NA	NA	SRG	Retro.	3	A	36	80–90	19	Y	Bilateral rupture (intracapsular)	
C11L	7.46E+05	67	85	SRG	Retro.	3	A	2	30–40	24	N	Cap con	-
C11R	7.42E+05	68	94	SRG	Retro.	1	A	2	30–40	24	N	Cap con	-
C12L	1.38E+05	157	249	SRS	Retro.	2	A	46	60–70	18	Y	Cap con	-
C12R	8.14E+05	46	57	SRS	Retro.	4	A	46	60–70	18	Y	Cap con	Calcified
C13R	1.90E+06	109	135	SRG	Retro.	3	A	2		23	Y	Cap con	-
C14L-A *	1.18E+06	108	133	SRG	Sub.	2	A	15	40–50	23	N	Lett rupture (intracapsular) / ptosis	IR; soft area
C14L-B *	1.04E+06	57	107	SRG	Sub.	2	A	15	40–50	23	N	Lett rupture (intracapsular) / ptosis	IR; calcified area
C14R	2.45E+06	41	-	SRG	Sub.	2	A	15	40–50	23	N	Lett rupture (intracapsular) / ptosis	Extracapsular rupture
C15L	2.21E+06	113	135	SRG	Retro.	2	A	8	30–40	23	N	Malposition / Ptosis	-
C15R	2.27E+06	42	49	SRG	Retro.	2	A	8	30–40	23	N	Malposition / Ptosis	-
C16L	2.88E+05	126	396	SRS	Retro.	2	A	24	60–70	22	N	Deflated implants	Deflated
C17L	4.41E+05	42	49	TAG	Sub.	2	A	9	30–40	23	N	Malrotation	Non-adherent
C17R	7.68E+05	218	664	TAG	Sub.	2	A	9	30–40	23	N	Malrotation	Non-adherent

†All capsules were collected from aesthetic surgery, except sample 1, which comes from a reconstructive surgery.

TAG = Textured Anatomical Gel; TRG = Textured Round Gel; SRG = Smooth Round Gel; SRS = Smooth Round Saline; Retro. = Retropectoral; Sub. = Subglandular; ER = Extracapsular Rupture; IR = Intracapsular Rupture.

*C14L-A & C14L-B were sourced from the same capsule, however a portion of the capsule was calcified so the capsule was subsampled into “soft” and “calcified” portions and treated separately. NA = No Amplicons.

Blank-corrected values are presented for both observed sequences and chao 1. Note that Baker scales I and II are considered non-pathologic, whereas III and IV are pathologic.

**Table 2:** Test of inhibition of 16S amplification by host tissue DNA using qPCR (see methods).

Sample	Spike (E. coli ng/uL)	Total copy number
E. coli	24.1	2.92E+10
E.coli	2.4	6.32E+08
E. coli	0.24	5.54E+07
C13R	-	5.35E+05
C13R	24.1	1.56E+08
C13R	2.4	1.35E+07
C13R	0.24	1.36E+06
C17L	-	3.35E+05
C17L	24.1	1.54E+08
C17L	2.4	1.31E+07
C17L	0.24	1.17E+06

Data is presented as total number of 16S copies.

Amplicon sequencing of 16S rRNA gene copies from low biomass samples can also be prone to contamination associated with library preparation. To evaluate such contamination in our data we sequenced both our extraction and PCR blanks, which mostly yielded sequences affiliated to the Proteobacteria, Firmicutes, and Actinobacteria phyla. To correct microbial community profiles for such possible contamination, we proportionally removed reads from samples based on the relative abundances of sequences recovered from our extraction blanks. The most abundant sequence in the blank was used to estimate the fraction of reads in a sample that could have resulted from contamination, conservatively assuming all sample reads with that sequence were derived from contamination. We then subtracted reads from other sequences based on their proportional abundance in the blank. This blank correction can be written as:

$$S_{corr}=S-S_{max}*\frac{B}{B_{max}}$$

Where *S*_*corr*_ is the blank corrected read count for a given sequence, *S* is the raw read count for the same sequence, *S*_*mas*_ is the sample read count of the most abundant sequence in the extraction blank (in this case *E*. *coli*), *B* is the read count for a given sequence in the blank, and *B*_*max*_ is the read count of the most abundant sequence in the blank. On average this blank correction decreased the number of observed sequences by 4%, and at a maximum, it decreased the number of observed sequences by 16%. We thus refer to this blank corrected data throughout the rest of the paper.

**Fig 1:**
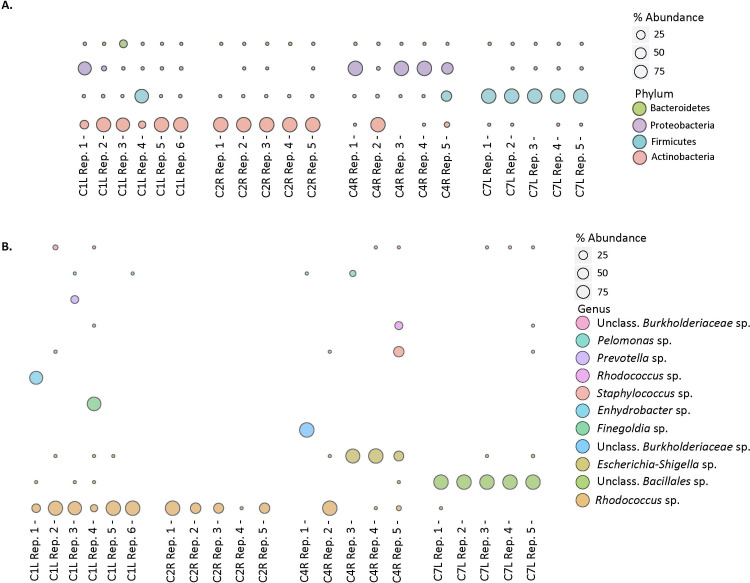
Microbial community composition. (a) Distribution of 16S rRNA sequences of the 4 most abundant phyla in replicate capsular samples. Bubble size per phylum represents the percentage of the total reads for each sample. (b) Distribution of the most abundant 16S rRNA sequences classified at the genus level, in replicate capsular samples. Bubbles size per genus represents the percentage of the total reads for each sample.

Replicate (5–6) analyses of microbial community composition were conducted on 4 contracted capsules, with a range in bacterial loads, and the degree of heterogeneity within a given contracted capsule was variable (Fig 1). Of the 4 capsules evaluated, 2 of these exhibited identical communities, at the phylum level, across 5 separate extractions (Fig 1A). Capsule C2R was largely comprised of Actinobacteria of the *Rhodococcus* species (sp.) (range 99.8–99.9%), while capsule C7L was largely comprised of Firmicutes of the unclassified Bacillales sp. (range 99.7–99.8%) (Fig 1B), across the 5 replicates analyzed. The 2 other capsules evaluated were somewhat heterogeneous across the separate extractions. For example, of the 6 replicate analyses of capsule C1L, 4 were dominated by members of the Actinobacteria, 1 by Firmicutes, and 1 by Proteobacteria. Appreciable Bacteroidetes were only present in 1 replicate (Fig 1A). Similarly, replicates of capsule 4R were also variable, and while 3 of the 5 replicates were exclusively Proteobacteria, 1 was exclusively Actinobacteria and the other was mixed Proteobacteria and Firmicutes. At the species level (97% identity in the 16S rRNA gene) capsule C1L was dominated by *Rhodococcus* sp. (13.2–99.6%), *Enhydrobacter* sp. (up to 77.6% in a single replicate), and *Finegoldia* sp. (up to 85.8% in a single replicate) across 6 separate replicates (Fig 1B). In capsule C4R, there was a larger degree of variation at the species level between replicates, with the most abundant OTUs being the *Escherichia-shigell*a sp. (0–99.8%), *Rhodococcus* sp. (0–99.1%), unclassified Burkholderiaceae sp. (0–99.6%), *Staphylococcus* sp. (41.0%) and *Acinetobacter* sp. (0–18.7%) (Fig 1B). Notably, both homogeneous capsules, C2R and C7L, had high bacterial loads, 10^6^−10^7^ 16S rRNA gene copies g^-1^, while the more heterogeneous capsules, C1L and C4R, had lower loads of 10^5^−10^6^ rRNA gene copies g^-1^ (Table 1).

**Fig 2:**
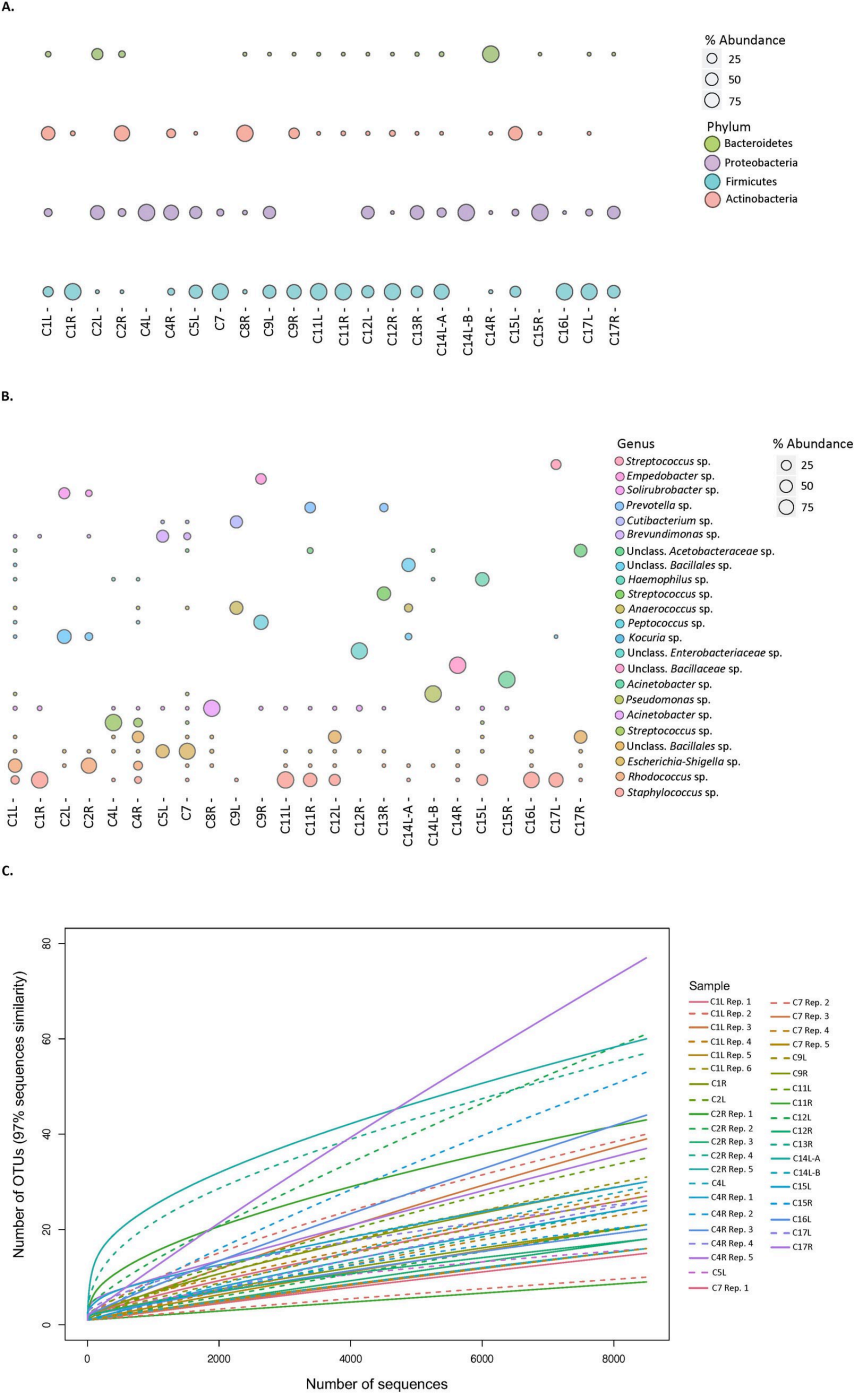
Microbial community composition. (a) Distribution of 16S rRNA sequences of the 4 most abundant phyla in capsular samples. Bubbles size per phylum represents the percentage of the total reads for each sample. (b) Distribution of the most abundant 16S rRNA sequences classified at the genus level, in capsular samples. Bubbles size per genus represents the percentage of the total reads for each sample. (c) Microbial diversity of breast capsule samples sequenced. Rarefaction curves are based on sequences with 97% sequence identity. Parentheses denote paired right and left breast samples. Note sample C8R and C14R were excluded from rarefaction analysis as the samples had half the number of total sequence reads compared to other samples.

Microbial community profiles based on 16S rRNA gene sequences recovered from a wider suite of contracted capsules comprised 3–4 principle phyla and 16–218 observed species (Fig 2A and 2B and Table 1). Rarefaction analyses revealed that resampling of the observed taxa approaches, but doesn’t quite reach, asymptotic values and thus may not capture the full diversity of the microbial communities (Fig 2C). Bacterial diversity metrics at the 97% sequence identity level estimate capsule microbial community taxonomic richness (Chao 1) of 24–664 species, or more (Table 1). Most capsule microbial community members belong to the Actinobacteria (0–99.9%), Firmicutes (0.01–99.9%), Proteobacteria (0–99.9%), and Bacteroidetes (0.01–17.3%) (Fig 2A). With a cutoff of >1% average abundance across all samples, 23 species remain (Fig 2B), and of these species, the top 6 most abundant represent a combined average of 49% of all sequences per sample, which were assigned to, *Staphylococcus* sp. (range 0–99% present in 15/24 samples), *Rhodococcus sp*. (range 0–83% present 15/24 samples), unclassified *Bacillales* sp. (range 0–92%, present in 17/24 samples), *Escherichia-shigella* sp. (range 0–48%, present in 9/24 samples), *Pseudomonas* sp. (range 0–99%, present 4/24 samples), and *Cutibacterium sp*. (0–98%, present in 16/24 samples) (Fig 2B). The other 18 species were present in a fewer number of samples, on average, and varied widely in abundance between samples (range 0–99%, present in <5/24 capsules).

**Fig 3:**
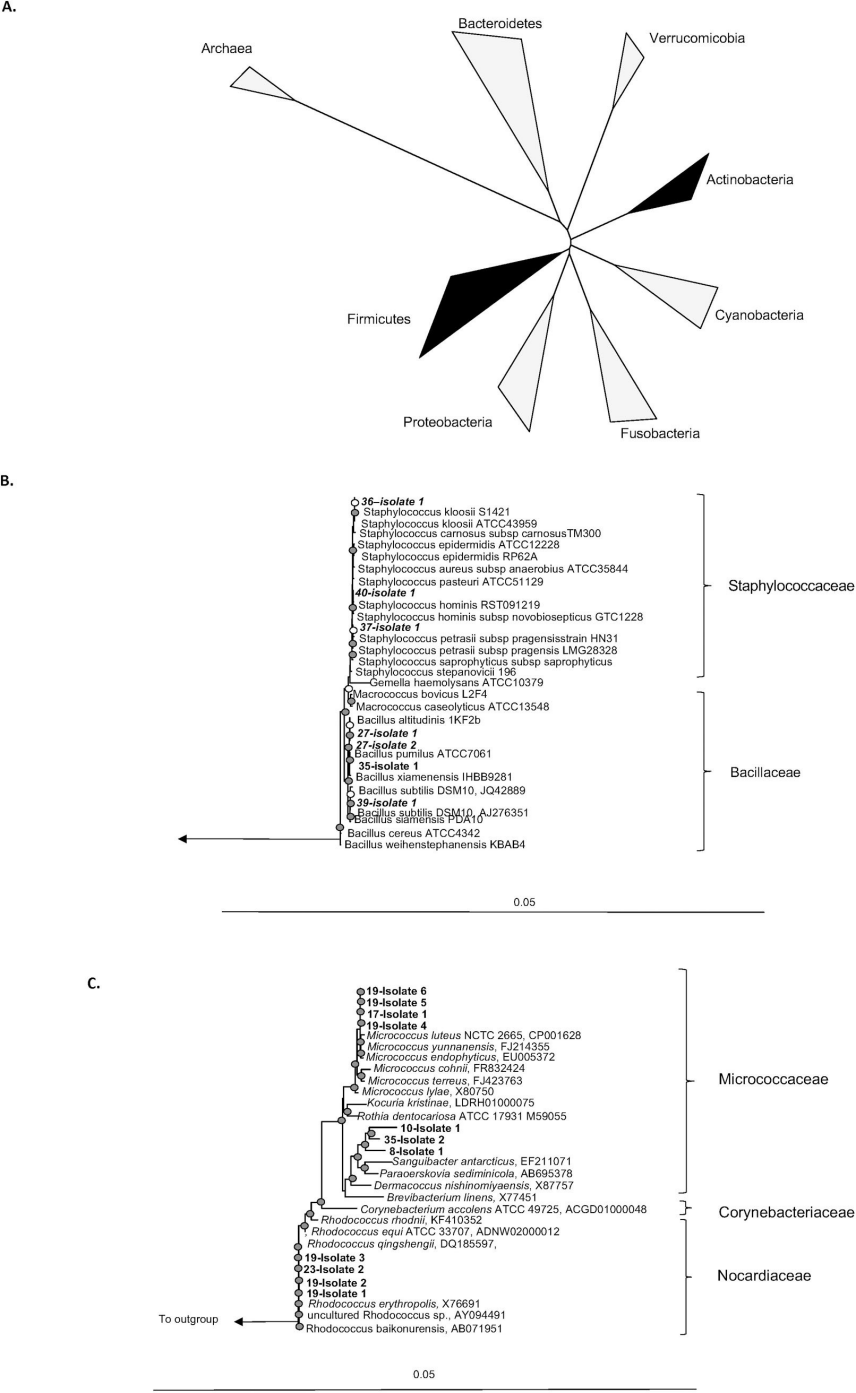
Bacterial isolates from capsule samples. (a) Bacteria commonly associated with human tissue. Phylogeny is based on the 16S rRNA gene. Dark wedges indicate bacteria we isolated from breast implant capsules; (b, c) Phylogeny of 16S rRNA gene sequences from our isolates (b) Firmicutes, (c) Actinobacteria. Displayed trees (b) and (c) were constructed in Arb using maximum likelihood. Filled circles indicate bootstrap support (maximum parsimony, with 100 resamplings) of ≥ 90%, and open circles represent ≥ 75% support. Bar, 5% sequence divergence.

To supplement our amplicon sequence data and initiate lab culture strains for follow-on physiological and genomic studies, we also isolated organisms from an arbitrarily selected subset of the capsules. Swabs of tissue or saline solutions remaining after tissue storage were streaked onto LB agar and incubated at 37°C. Pure isolates were selected for 16S rRNA gene sequencing to describe their taxonomy and relate the isolates to the cultivation-independent characterization of the capsule communities. Our isolate collection is comprised exclusively of members of the Firmicutes and Actinobacteria (Fig 3A). At higher taxonomic resolution, our isolates were identified as *Rhodococcus* sp., *Bacillus* sp., *Staphylococcus* sp., and *Micrococcus* sp. (Fig 3B and 3C).

**Fig 4:**
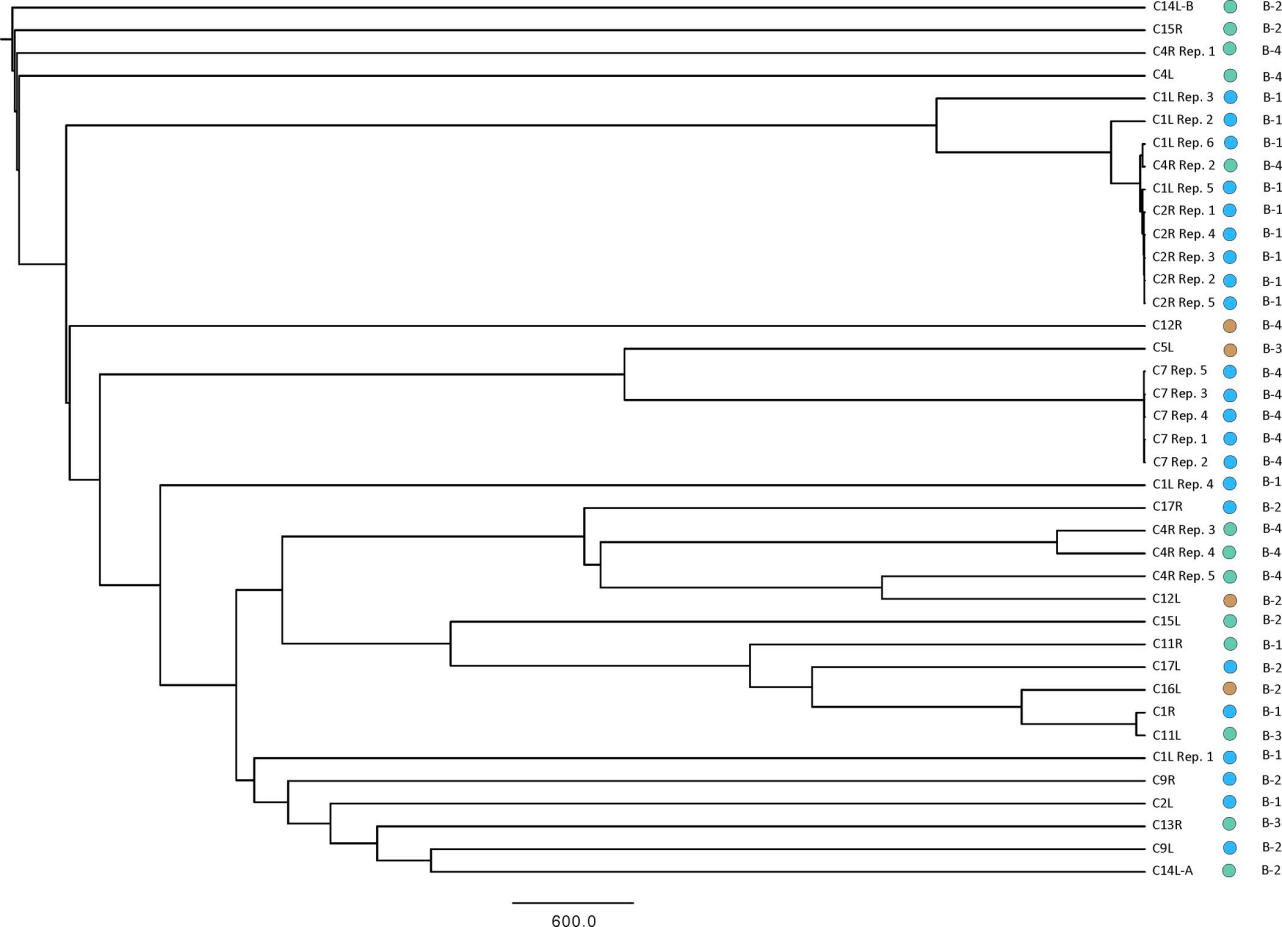
Hierarchical clustering analysis. Hierarchical relationship among samples based on Euclidean distance of 16S- OTU abundances. The UPGMA clustering algorithm was used to obtain the hierarchical relationships between samples. Node labels indicate the sample ID. Note sample C8R and C14R were excluded from rarefaction analysis as the samples had half the number of total sequence reads compared to other samples. Circle color: Green = Smooth Round Gel; Blue = Textured Gel (round or anatomical); Brown = Smooth Round Saline. B stands for Baker.

Microbial community profiles were compared to a limited number of variables related to implant type and degree of capsular contracture graded through the Baker scale. The Baker scale is defined as: Grade I in which the breast is normally soft and appears natural in size and shape; Grade II in which the breast is a little firm, but appears normal; Grade III in which the breast is firm and appears abnormal; and Grade IV in which the breast is hard, painful to the touch, and appears abnormal [37]. Baker Grade I and II capsules are not pathologic, while III and IV grades are more strongly contracted and therefore pathologic. A hierarchical clustering analysis was used to test for relationships between implant type, plane of placement, and Baker contracture grade (Fig 4).

## Discussion

In our study, more than 80% of capsules analyzed had amplifiable microbial 16S rRNA genes, which is comparable to, or higher than observations made in previous amplicon-based studies of capsular contracture (42% [30], and 8% [31]). Differences observed across studies likely reflect variations in methodological approaches. In particular, our use of nested PCR amplification to overcome PCR inhibitors from host material likely enhanced detectability in our study. Our documentation of strong PCR inhibition suggests that even appreciable bacterial loads may go undetected through standard amplicon-based analyses. Furthermore, our observations of variability, even at the scale of individual capsules, may ultimately manifest as differences in detectability across studies. We also note that careful contamination control is key for enabling robust microbiome detection.

Bacterial loads in contracted capsules ranged from 10^5^ to 10^7^ 16S rRNA gene copies g^-1^ of capsular tissue. While 3 orders of magnitude lower than previous analyses of breast tissues [24], the bacterial loads studied here are much higher than our blanks and we can thus rule out reagent contamination, which is known to confound microbial community profiling based on amplicon sequencing, particularly in low biomass samples [38]. The reason for the much lower bacterial loads observed here is uncertain, but may be related to inhibition in our qPCR analyses, as discussed above. Nevertheless, higher bacterial loads were generally associated with relatively homogeneous bacterial communities across replicates of the same sample, though diversity estimates (chao1) appeared unrelated to bacterial load between samples (Table 1). A larger number of capsule community analyses would be required to properly test these relationships, or lack thereof.

Microbial communities associated with contracted capsules are comprised of a few relatively abundant community members that are broadly distributed across multiple capsules, as well as a broad suite highly variable, low abundance members. Microbial communities associated with contracted capsules are comprised of 24–664 species (chao 1) and are similar in diversity to healthy breast tissue (121 taxa [39], 125–130 [40]) but far less diverse than, for example the, 10^3^ species commonly found in the human gut [41], 10^4^ species on human skin [42], or the >10^4^ species common in soils [43]. Our analyses suggest that capsule microbiomes are more diverse than what previous amplicon-based sequencing observations suggest, given that these studies found on average only 8 bacterial species per capsule biofilm [30]. Again, these differences likely reflect methodological variation and could be related to DNA extraction, the region of the 16S rRNA gene targeted for amplification, or depth of sequence. In our study, most capsule community members belong to the Actinobacteria, Firmicutes, and Proteobacteria, with a minor component from the Bacteroidetes (Fig 2A). These same four phyla were previously recovered from breast tissues removed from healthy women and women with benign and malignant tumors [39, 44–46], as well as from heavily contracted capsules [30], including those associated with BIA ALCL [47]. Analyses at higher taxonomic resolution (genus level) show that while 20% of the capsule microbial communities were dominated by *Staphylococcus sp*., the remaining capsule community members comprised 4 additional principle taxa that were both greater than 1% average abundance across all capsules and present in more than 40% of the capsules studied. These belong to the Firmicutes, Proteobacteria, and Actinobacteria, demonstrating the importance of multiple other taxonomic groups to capsule communities. *Staphylococcus* sp. are frequently cultured from contracted capsules [18, 19] and are indeed prevalent (15 of 24) in the capsules we analyzed through amplicon sequencing, but many of the other abundant species have not been widely reported in previous culture-based studies of contracted capsules, illustrating the utility of culture-independent analyses. The relative prevalence of many of these predominant taxa across all samples collected implies that these taxa likely represent common implant colonizers with potential for biofilm formation. Of the species with >1% average abundance across all samples, some have been reported as skin commensals like the *Staphylococcus* sp. [42, 48], but many others including; *Rhodococcus sp*., *Bacillales sp*., *Cutibacterium* sp., *Haemophilus* sp., *Kocuria* sp., *Finegoldia* sp., and *Escherichia-shigella sp*., are not commonly reported as part of the healthy skin microbiome [48, 49] and instead may be opportunistic pathogens [50–53]. The majority of the species present below 1% abundance varied widely between samples and also within the same patient between left and right capsules. These microbial community members tend to be overwhelmed by the more common and abundant community members when bacterial loads are qualitatively high. This suggests that lower sensitivity analyses with partial DNA extraction yields, strong PCR inhibition, or shallow depth of sequence, might miss important biofilm community members. Our data implies that in addition to the group of highly abundant Firmicutes, Proteobacteria, and Actinobacteria, capsule communities consist of highly variable low relative abundance members.

Our isolate collection is comprised exclusively of members of the Firmicutes and Actinobacteria, missing entirely the Proteobacteria and Bacteroidetes detected through culture-independent community profiling, and revealing the expected bias in the organisms cultivated. At higher taxonomic resolution, our isolates were identified as *Rhodococcus sp*., *Bacillus sp*., *Staphylococcus sp*., and *Micrococcus sp*. With the exception of the *Rhodococcus sp*. (Fig 3B and 3C), organisms from these genera have been detected in previous cultivation-based studies. Notably, *Rhodococcus* species are the most abundant member of the capsule community from C2R, from which it was isolated, giving confidence that some relevant, yet new, organisms can be brought under laboratory culture using enrichment-isolation approaches standard to classical microbiology.

To compare our isolates to other studies with greater resolution, we conducted phylogenetic analyses of the full-length 16S rRNA genes recovered from our isolates. These phylogenetic analyses reveal that even cultivation-based approaches recover a diversity of organisms distinct at the species level (97% identity in the 16S rRNA gene). Organisms of the same species are well known to exhibit differences in genomic composition and metabolic potential—*Escherichia coli* is a classic example with up to 60% non-redundant genomic information across 3 ecologically diverse strains [54]. Notably, none of our isolates belong to the *S*. *epidermidis* or *S*. *aureus* species commonly implicated in capsular contracture [22, 35, 36]. Instead, our isolates are distributed across the *Staphylococcus* genus including organisms most closely related to *S*. *hominis*, *S*. *kloosii*, and *S*. *petrasii* sp. that can be associated with the human skin microbiome [42], but also have been implicated in pathogeneses [55, 56]. These isolates may thus provide systems with which to interrogate microbial physiology relevant to capsule biofilm formation and contracture.

We evaluated relationships between implant type, plane of placement, Baker contracture grade, and microbial community composition through hierarchical clustering analyses (Fig 4). In hierarchical clustering, differences in microbial community composition between groupings result in branching patterns whereby capsules from a specific individual group cluster together. Capsules recovered from the left and right breasts of the same patient provide us an opportunity to assess variability within an individual patient. Importantly, replicate samples of the same capsule tend to cluster together, particularly for C7, C2R, and C1L, with C4R exhibiting more spread. Importantly, left and right capsules do not cluster together, which implies that the microbial communities in the left and right capsules are distinct and likely not regulated by the same factors, like patient history or skin microbiome. Microbial community composition, furthermore, does not tend to cluster according to implant type or Baker scale of capsular contracture, suggesting that other variables may also contribute to determining the specific composition of microbial communities associated with contracted capsules. These observations should be tempered given the limited number of observations in the current study and improved statistics from a larger number of patients may help reveal relationships between microbial community composition, implant type, and capsular contracture.

## Conclusions

Our analyses reveal that microbial communities associated with pathologic and non-pathologic contracted capsules are considerably more diverse than previously appreciated. Taxa from 4 main phyla- the Proteobacteria, Actinobacteria, Firmicutes, and Bacteroidetes- are most commonly present and at appreciable relative abundances. This observation is consistent across multiple other cultivation dependent and cultivation independent studies. By contrast, however, we also find a number of other taxa that are generally present at variable and low relative abundances that sometimes can represent appreciable fractions of the community. This result appears to contrast with previous studies, but reveals that methodological variability across studies may lead to differing results and conclusions. This highlights a need for the cosmetic surgery community to develop standards of best practice if amplicon sequencing approaches are to be adopted in research and clinical practice, more broadly. In particular, these standards should: 1) address contamination; 2) select regions of the 16S rRNA or other genes to be used; 3) optimize DNA extraction and amplification protocols; 4) establish minimum information standards for metadata; and 5) develop consensus on sequencing platforms and depth. Our findings, that capsule associated microbial communities are diverse and highly variable, suggests that mitigation of microbial capsule colonization could be best addressed through personized approaches that consider the broader patient microbiome. As noted above, however, standards of best practice across the community could promote further research, by multiple groups, that would be needed to dramatically increase the number of observations and provide the statistical power needed to establish cause and effect relationships.

## Material and methods

### Ethical approval

Ethics approval for this study was obtained through the University of British Columbia Clinical Research Ethics Board under “Biofilm Study” H16-01002.

### Sample collection

Capsule samples analysed in this study were all collected in the private practice of author NJC from patients that underwent revision surgery consisting of implant exchange or removal. The sample series represents 17 consecutive cases between February of 2015 and June of 2016. All capsule specimens were collected from aesthetic patients except capsule 1, which was collected from a reconstruction surgery. Given that a goal of this study was to examine biofilm composition across a range of pathological and non-pathological capsules, we analysed a suite of samples that varied from Baker grade I to IV. Reasons for surgery included implant malposition, implant rupture, pathological capsular contracture, or patient desire to change implant type. Capsular tissue samples were removed aseptically from the patient and placed separately in sterile containers, containing saline. In most cases one sample was taken from a visually representative area of the capsule of each implant. In one case, C14, an additional sample was taken from one of the implant capsules because of the presence of the distinctly different appearance in areas of the capsule surrounding the implant. The implants were processed for microbiological analysis within approximately 24 hours of removal.

### Cultivation and isolation

Aseptically cut tissue sections were swabbed for culturing onto LB agar. Plates were incubated at 37°C for 1–7 days. Pure colonies selected for sequencing were grown up overnight in LB broth 37°C, then lysed at 95 ^∘^C for 30 minutes. 1 μl of lysed sample was used in the PCR with the universal primers 27F/1492R, to amplify full-length bacterial 16SrRNA genes. PCR amplifications were carried out with the following cycling conditions; initial denaturing at 95 ^∘^C for 3 min, 30 cycles of 95 ^∘^C for 30 s, 48 ^∘^C for 30 s, and 72 ^∘^C for 60 s, followed by a final elongation step at 72 ^∘^C for 10 min. PCR products were Sanger sequenced with forward and reverse primers (27F/1492R) by GeneWiz LLC.

### Sample processing and DNA extraction

Microbial DNA was extracted from approximately 0.25 g of breast capsular tissue using the Mobio PowerMax^®^ Soil DNA Isolation Kit, as per manufacturer’s instructions. Resulting DNA was stored at -20 ^∘^C. The quality and quantity of genomic DNA were measured on a NanoDrop® ND-1000 spectrophotometer (Thermo Scientific) and by PicoGreen (Quant-iT dsDNA kit, Invitrogen).

### SSU rRNA gene amplification and iTag sequencing

Bacterial and archaeal 16S rRNA gene fragments from the extracted genomic DNA were amplified using primers 515F and 806R. Sample preparation for amplicon sequencing was performed as described as [57]. In brief, the aforementioned 16S rRNA gene-targeting primers, complete with Illumina adapter, an 8-nt index sequence, a 10-nt pad sequence, a 2-nt linker and the gene specific primer were used in equimolar concentrations together with dNTPs, PCR buffer, MgCl, 2 U/μl high fidelity Platinum Taq DNA polymerase and PCR-certified water to a final volume of 50 μL. PCR amplification was performed with an initial denaturing step of 95 ^∘^C for 2 min, followed by 30 cycles of denaturation (95 ^∘^C for 20 s), annealing (55 ^∘^C for 15 s), and elongation (72 ^∘^C for 5 min), with a final elongation step at 72 ^∘^C for 10 min. A nested-PCR approach, using primers 27F and 1492R for the initial amplification, followed by a second amplification using primer pair 515F/806R under the conditions outlined above, was adopted for samples in which the original PCR did not yield any products. Equimolar concentrations of prepared amplicon samples were pooled into a single library by using the Invitrogen SequalPrep kit. The amplicon library was analysed on an Agilent Bioanalyzer using the High Sensitivity dsDNA assay to determine approximate library fragment size, and to verify library integrity. Pooled library concentration was determined using the KAPA Library Quantification Kit for Illumina. Library pools were diluted to 4 nM and denatured into single strands using fresh 0.2 N NaOH as recommended by Illumina. The final library was loaded at a concentration of 8 pM, with an additional PhiX spike-in of 5–20%. Sequencing was conducted at the University of British Columbia Sequencing and Bioinformatics Consortium (http://sequencing.ubc.ca) All raw sequence data (including blanks) were submitted to the SRA database under accession PRJNA632935.

Bacterial DNA was quantified by quantitative polymerase-chain reaction (qPCR) using the SsoFast™ EvaGreen assay (Bio-Rad™) and a CFX96 Real-Time Detection System (Bio-Rad™). The 16S rRNA gene was targeted using bacterial-specific primers, 27F, (5′-AGAGTTTGATCCTGGCTCAG) and DW519R (5′-GNTT TACCGCGGCKGCTG). Each amplification reaction (20 μL) contained PCR certified water (4μL), SsoFast™ EvaGreen master-mix (10 μL), and 3 μM primer (2 μL). Amplification of the 16S rRNA gene was performed with an initial denaturation step (95°C, 3 min), followed by 45 cycles of: denaturation (95°C, 20s); annealing (55°C, 30s); and elongation (72°C, 30s). This was followed by a melt curve analysis for assessing amplicon specificity (ramp-up of 0.5°C every second, increasing from 55°C to 95°C). Bacterial DNA standards, ranging from 10^2^−10^8^, non-template controls, and sample DNA were all run in duplicate. The qPCR assays were tested for inhibition by adding known copies of E. Coli 16S rRNA genes and comparing the resulting measured copy numbers to the expected copy numbers.

### 16S rRNA amplicon sequence data analysis and visualization

Sequences were processed using Mothur and the Miseq protocol [58]. Sequences were removed from the analysis if they contained ambiguous characters, had homopolymers longer than 8 bp, and did not aligned to a reference alignment of the correct sequencing region. Unique sequences and their frequency in each sample were identified, and a pre-clustering algorithm was used to further de-noise sequences within each sample [59]. Unique sequences were identified and aligned against a SILVA (v.132) alignment (available at http://www.mothur.org/wiki/Silva_reference_alignment). Sequences were chimera checked using VSEARCH [60] and reads were clustered into 97% OTUs based on uncorrected pairwise distance matrices. OTUs were classified using SILVA reference taxonomy database (release v.132, available at http://www.mothur.org/wiki/Silva_reference_files). All data was visualized in RStudio (RStudio Team, 2015) [61]. For generation of Chao1 and Hierarchical clustering the number of reads per sample was rarefied to 8494 sequences per sample. Samples C8R and C14R were excluded from these analyses as their read number was lower (4848 and 5099 respectively).

### Full-length 16S analysis

Full-length 16S rRNA sequences were quality checked using the Sequencher software package (version5.2, GeneCodes Corporation, Ann Arbor, MIUSA http://www.genecodes.com). 16S rRNA gene sequences were compared to available databases using the Basic Local Alignment Search Tool (BLAST) [62]. To determine approximate phylogenetic affiliations, sequences were aligned using the SINA Web Aligner [63] then imported into the ARB programme package for manual editing using the SILVA database [64]. All subsequent phylogenetic analyses were performed in ARB [65]. Maximum likelihood algorithms were used to calculate a phylogenetic tree, with maximum parsimony-based bootstraps (100 re-samplings) also calculated to assess the stability of observed branching patterns.
